# Activation and Switching of Supramolecular Chemical Signals in Multi-Output Microfluidic Devices

**DOI:** 10.3390/mi13101778

**Published:** 2022-10-19

**Authors:** Artem Bezrukov, Yury Galyametdinov

**Affiliations:** Department of Physical and Colloid Chemistry, Kazan National Research Technological University, Kazan 420015, Russia

**Keywords:** microfluidics, chemical signal, polyelectrolyte, surfactant, reaction front

## Abstract

In this study, we report on the developing of a continuous microfluidic reaction device that allows selective activation of polyelectrolyte-surfactant chemical signals in microflows and switches them between multiple outputs. A numerical model was developed for convection-diffusion reaction processes in reactive polymer-colloid microfluidic flows. Matlab scripts and scaling laws were developed for this model to predict reaction initiation and completion conditions in microfluidic devices and the location of the reaction front. The model allows the optimization of microfluidic device geometry and the setting of operation modes that provide release of the reaction product through specific outputs. Representing a chemical signal, polyelectrolyte-surfactant reaction products create various logic gate states at microfluidic chip outputs. Such systems may have potential as biochemical signal transmitters in organ-on-chip applications or chemical logic gates in cascaded microfluidic devices.

## 1. Introduction

Microfluidics is a vibrant interdisciplinary area of fundamental and applied research that bridges computing and chemistry by offering fluidic microdevices that process chemical signals [1,2,3,4], create chemical transistors [5] and generate chemical logic gates [6,7,8,9,10]. Microfluidic chips offer capabilities for processing chemical signals represented by soft matter and biomolecular systems [1,3,11,12]. They are particularly attractive as automated analysis tools for biological and medical applications, such as detection of cellular responses and gene expression [1], sensing glucose and insulin [11], or studying cell dynamics [3]. Processing of chemical signals is essential for proper functioning of microfluidic organ-on-chip prototypes. such as hearts, lungs, or guts. [13,14,15] or on-chip models of skin or endocrine systems [16,17].

Polymers and colloids are promising candidates for soft matter chemical signal transmission in biomedical applications because they represent biomolecular systems (proteins, nucleic acids, polysaccharides, or lipids) or their models [18,19,20,21,22,23,24]. A microfluidic potential of polymer and colloid chemical signals is, however, far from unleashing its full capacity. Current studies in the field of microfluidic chemical signals and logic gates focus mostly on low-molecular compounds and microscale droplets [2,6,7,8,9,25,26,27].

In their turn, polymers and colloids possess an intrinsic capability of being signal transmitters in microfluidics. as their interaction products are extremely sensitive to a variety of factors, such as concentration and ratio of components [22,28,29], solvent [30], pH [31], among others. Nonequilibrium microfluidic confinement allows the simultaneous application of these factors to synthesize and modify soft matter structures [32,33,34,35,36]. By varying microchip operation parameters, we can use various mixing strategies for polymers, colloids and related low-molecular compounds, such as electrolytes [35,37,38,39,40]. It Is possible, therefore, to control the behavior of reactive soft matter systems in microchannels and vary the size, dispersity, or concentration fields of the reaction products in a broad range [32,38,41,42,43,44]. It is a potent strategy for on-demand generation of various supramolecular chemical signals in microchips. In addition, the application focus of microfluidic soft matter systems is biomedicine [20,38,41,45,46,47,48] that matches the application trends of microfluidic chemical signal and logic gate circuits.

Thus, revealing a correlation between the operation parameters of microfluidic chips, the behavior of reactive polymer and colloid systems, and the resulting properties, and concentration distributions of their interaction products, is of an urgent research interest. The correlation is essential to discovering novel approaches to developing microscale chemical signal generators, which can be components of supramolecular chemical processors.

This work aimed at formalizing the operation parameters of microfluidic reactors that allow precise control of the distribution of polyelectrolyte-surfactant reaction products in confinement and the selective switching of these between multiple outputs. We combine these parameters into the scaling laws and set the threshold values of the respective similarity criteria to predict initiation and completion of the polyelectrolyte-surfactant complexation reaction in microfluidic chips. The reaction products are 100 nm nanoparticles, which represent an easily detectable chemical signal. Numerical simulations and experimental verifications proved that we could activate the microfluidic reactor on demand and switch the chemical signal between multiple outputs to create various logic gate states.

## 2. Materials and Methods

### 2.1. Materials

Polydiallyldimethylammonium chloride (PDADMAC) was purchased from Sigma Aldrich (St. Louis, MO, USA). The polymer was sold as a viscous liquid (20% aqueous solution) and was used as received. Sodium dodecyl sulfate (SDS) was purchased from BDH Limited, Poole, England, and used as received. CTAB and SDS were sold as a powder.

Deionized water (18.2 MΩ-cm) was used for all solutions. Before preparing solutions, water was filtered by 0.2 µm Millipore PTFE filters.

The test reaction was the association of a cationic polyelectrolyte polydiallyldimethylammonium chloride (PDADMAC) with sodium dodecyl sulfate (SDS). We selected these materials as model substances because their macroscopic associations and phase behaviors are well-characterized [22,28,29]. In the literature we found SDS diffusion coefficients so as to estimate polyelectrolyte-surfactant association dynamics and rate constants [49,50,51,52]. These substances are convenient for numerical simulations and ensure a smooth comparison of experimental and numerical data.

Polydimethylsiloxane (PDMS) Sylgard 184 was purchased from Dow Corning (Midland, MI, USA) and used to fabricate microfluidic devices. It came as a two-part elastomer kit (the pre-polymer and curing agent). SU-8 3050 photoresist (Microchem Corp., Westborough, MA, USA) was used to produce a mold for microfluidic chips.

### 2.2. Solutions and Microfluidic Experiments

For microfluidic experiments, bulk samples of 1 g/L PDADMAC were produced from their initial solutions and allowed to dissolve overnight. An amount of 50 mmol/L KCl was used as a background electrolyte. The concentration of monomers in 1 g/l PDADMAC solution was 6.1 × 10^−3^ mol/L. Bulk samples of 6.1 × 10^−3^ mol/L SDS were produced by dissolving dry surfactant in deionized water. This concentration of SDS is below its critical micelle concentration, which is reported to be about 8.4 mmol/L [52].

The concentrations of polymers and surfactants in all the solutions were low enough to avoid considerable differences between the viscosities of miscible flows in microchannels.

PDADMAC and SDS solutions were infused into microfluidic devices by Harvard Apparatus 2000 syringe pumps. The microfluidic set-up was designed to assure the same flow path conditions for all the fluids to the main channel of the microfluidic device. We used two identical syringe pumps and the same syringes with equal initial volumes of the reagents and solvent. Two syringes with the reacting solutions were installed into the first pump to provide identical flow rates of the reagents in all the experiments. The syringe with the solvent was installed into the same second pump, so its flow rate could be varied independently. All the liquids were fed into the chips through PTFE tubes of the same length (10 cm) and internal diameters that fit the needle tips inserted into microchip inlets (20 G-type needles, 0.9 mm diameter). All the inlet microchannels in all the chips were designed to have the same length and width.

The flow rates of polymer and surfactant solutions and a solvent were varied in the range of 0.1–10 µL/min. The flow rates of PDADMAC and SDS solutions were set equal in all the experiments and provided equal volume-to-volume ratios of these reagents. The duration of all the microfluidic experiments was 15 min.

### 2.3. Methods

Hydrodynamic diameter PDADMAC macromolecules and PDADMAC-SDS complexes were measured using the Malvern Zetasizer Nano ZS light scattering system. Hydrodynamic sizes and diffusion coefficients corresponding to the maximum of the number-average size distribution of particles were used in simulations and experiments. All the DLS measurements were repeated at least three times to obtain reproducible results.

Images were recorded on a Levenhuk D320 optical microscope. Microchannels were imaged at 10× magnification using a Levenhuk M1400 Plus camera with a resolution of 0.27 µm/pixel.

Convection-diffusion reaction equations for reacting polyelectrolyte-surfactant flows were solved using Matlab 2021a software with Partial Differential Equations Toolbox. Diffusion coefficients of SDS ions were obtained from [48]. For simulations, the value of the polyelectrolyte-surfactant association rate constant was set to 10^4^ L·mol^−1^·s^−1^.

### 2.4. Device Fabrication

Microfluidic devices were fabricated using standard photolithography techniques [52]. SU-8 photoresist and a transparency photomask with the negative image of a microchip were used to produce a 100 µm thick mold of microfluidic chips on top of a 3-inch silicon wafer. Sylgard 184 PDMS pre-polymer was mixed with a curing agent, poured over the mold, and allowed to cure for 4 h in a 60 °C oven. Once cured, PDMS was peeled off the mold and bonded to a flat PDMS slab via plasma treatment. The PDMS device was then heated in an oven at 180 °C for 1 h to finalize bonding of the two polymer layers.

## 3. Results

### 3.1. Designing Microfluidic Devices for Supramolecular Chemical Signal Generation

**Figure 1:**
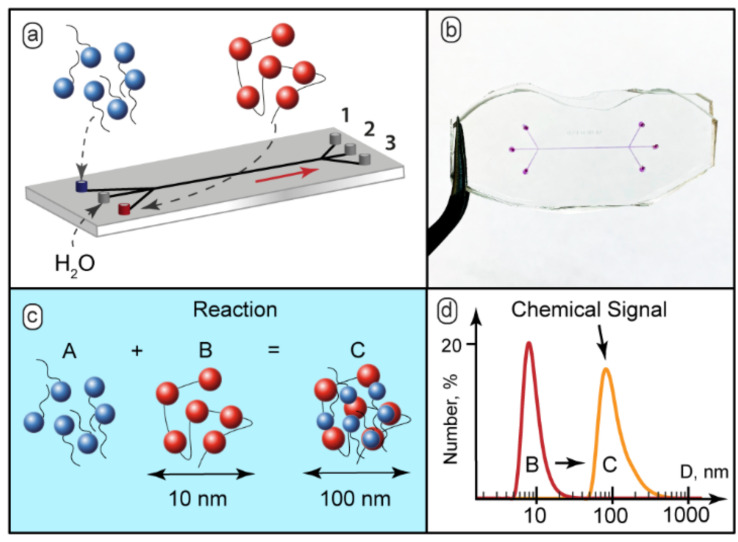
(**a**) Schematic diagram of feeding the reacting solutions and the solvent into a microfluidic chip and separating the reaction mixture into 3 streams; (**b**) The 3-input and 3-output microfluidic chips with width = 200 µm and length = 15 mm, the microchannels were filled with dye (phenolphthalein in 0.1 M KOH) to aid visualization; (**c**) SDS binding by PDADMAC macroions; (**d**) Chemical signal detection by DLS: initial 10–15 nm polymers macromolecules or smaller particles and 100–150 nm aggregates of complexes. The SDS:PDADMAC molar ratio in the test reaction was 0.2:1.

In microchips with contacting reacting flows, the reaction starts immediately after the junction of the input channels [53]. To avoid an immediate start of a chemical reaction, and add the on/off capabilities to our microfluidic devices, we separated reacting flows by a varied central flow of solvent by using devices with three inputs. Figure 1a demonstrates the schematic diagram of such a microfluidic device. Figure 1b shows the microfluidic chip prototype fabricated for the experiments. The microchannels were filled with dye for better visualization. The widths of all the channels were 200–300 µm. The length of the main channel was 15 mm. In the experiments, we also used 100 mm long chips. Variable lengths allowed convenient control of the residence time of the reagents in the main channel.

The complexation scenario of ionic polyelectrolytes with oppositely charged surfactants assumes formation of complexes with surfactant ions bound electrostatically to macromolecular sites [28] (Figure 1c). Bound surfactant ions reduce solubility of polyelectrolyte macromolecules, so they tend to organize into larger aggregates and, finally, precipitate [29].

The behavior of the PDADMAC-SDS system in macroscopic conditions was characterized by DLS. The DLS analysis revealed that the hydrodynamic diameter of individual polyelectrolyte macromolecules in the presence of 50 mmol KCl, as a background electrolyte, was about 10–15 nm (Figure 1d). The solutions with the SDS-to-PDADMAC molar ratio of 0.1–0.5 became turbid and contained particles with the hydrodynamic diameter of 100–150 nm (Figure 1d). With the SDS-to-PDADMAC molar ratio above 0.5, precipitates formed and the resulting transparent solution contained no nanoscale or sub-microscale particles.

For further microfluidic experiments, therefore, we used 6.1 × 10^−3^ mol/L PDADMAC solution and 1.2 × 10^−3^ mol/L SDS solution to provide a surfactant-to-polymer molar ratio of 0.2, which did not lead to precipitation in bulk.

The detected 100–150 nm particles were supposed to be aggregates of macromolecules that contained bound surfactant ions. DLS is sensitive to these aggregates and provided reliable detection results for concentrations of the reagents in an order of magnitude lower than the initial ones (6.1 × 10^−4^ mol/L). The size of aggregates gradually reduced with dilution (to 70–100 nm) but was still clearly different from the size of initial macromolecules.

Aggregated macromolecules represent a convenient chemical signal. This signal is easy to detect, even at low concentrations of reacting species. The size of the aggregates differed significantly from the hydrodynamic diameters of the original polymer macromolecules (about 10–15 nm according to DLS) or surfactant molecules (about 2–3 nm according to NMR [51]).

For the implementing of the chemical logic gate potential of microfluidic chips, we fabricated and studied microfluidic reactors with two and three outputs. In principle, two outputs are sufficient for achieving multiple logic gate capabilities. Such designs are common for commercial H-sensor microfluidic devices. We performed preliminary microfluidic tests with three-input and two-output devices. At relatively high flow rates of the reagents and the solvent (>20 µL/min), and wide solvent flows (0.6–0.9 of the main channel width), dynamic light scattering detected particles of no more than 10–15 nm in the output flows. In such flow conditions, polymer and surfactant molecules might not reach each other by diffusion, and their flows might turn out to release through different outputs without reacting. At lower flow rates (0.1–10 µL/min) and narrower solvent flow widths (<0.5), 100–150 nm particles were detected in both outputs in nearly all flow conditions. This effect might be associated with the formation of polymer-surfactant complexes in the center of the main channel and its distribution between two outputs. With two-output chips, therefore, we could achieve only on-off states of microfluidic devices without reproducible switching of a chemical signal (polymer-surfactant complexes) between the outputs, which is required for creating various logic gate states in a microfluidic chip.

With three output designs, we could achieve the same on-off states as in two-output devices at high flow rates and solvent flow widths. However, DLS analysis of samples collected at lower flow rates of the reagents and the solvent (0.1–10 µL/min) detected 100–150 nm particles in the central output or a combination of two or three outputs. Therefore, we could assume that three outputs might provide more options for chemical signal activation and switching, as compared with two-output geometries.

In further microfluidic experiments, the samples were collected separately at all 3 microchannel outputs and analyzed by DLS. Based on macroscopic characterization results, <15 nm for individual polymer macromolecules and >70 nm for aggregates, we selected a 50 nm diameter as the threshold value. The absence of particles above 50 nm in diameter in the collected samples was considered as “0” or the logical “False”. The presence of larger particles was considered as “1” or the logical “True”.

### 3.2. Modeling Microfluidic Polyelectrolyte-Surfactant Reactions in 3-Input and 3-Output Chips

To predict the reaction initiation conditions and the complexation product release through specific microchip outputs, we developed scaling laws that evaluate polyelectrolyte-surfactant complexation conditions in a microfluidic device with a central flow of buffer.

**Figure 2:**
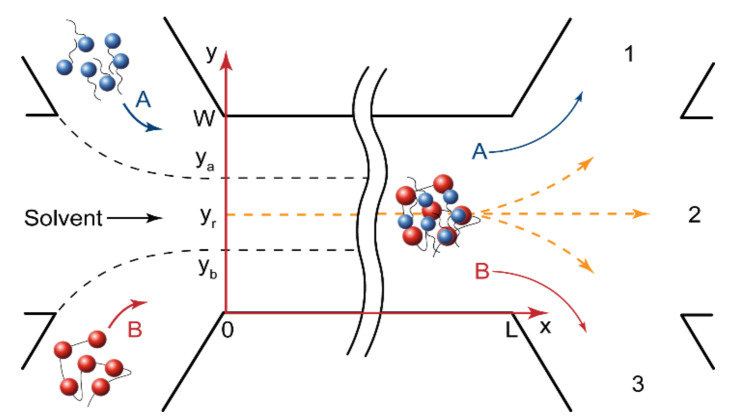
Geometry of microfluidic devices. The reagents were fed through the side input channels, the solvent was fed through the central input channel to avoid an immediate mixing of reagents and the start of reaction. The product formed at a distance from the junction of the input channels and then was distributed between output channels, depending on the reaction front position.

Figure 2 shows the geometry of a microfluidic chip with the width W and the length L. The central solvent flow prevented the A and B reactive flows from having an immediate contact and, therefore, from an immediate reaction start. The flow rates of polymer and surfactant were always equal, Q_A_ = Q_B_, to reduce the number of microfluidic pumps that were required for the device’s operation. The solvent flow could also be an additional control factor for performing reactions in microchips.

In the central solvent flow (Figure 2), the reaction started when the reacting molecules diffused from their flow boundaries (y_a_ and y_b_) to the site of reaction (y_r_). For a rectangular microchannel geometry (U_max_ = 3/2U), shown in Figure 2, the initiation time t_in_ required for a certain part of the reagent “A” molecules to reach the reaction site from the “A” flow was set as follows [53]:

(1) $$t_{\mathrm{in}}=\frac{{\left(y_{a}-y_{r}\right)}^{2}}{4D_{a}}=\frac{{\left(y_{r}-y_{b}\right)}^{2}}{4D_{b}}\approx \frac{L}{3/2U}$$

where U is the flow velocity; L is the microchannel length; D_a_ and D_b_ are the diffusivities of the reaction species; y_a_ and y_b_ are the boundaries of surfactant and polymer flow, respectively; y_r_ is the reaction front coordinate.

By introducing a set of dimensionless numbers, we could transform Equation (2) into the scaling law for the reaction initiation at the main channel end (x = L) and predict the reaction front location. These numbers were Peclet number Pe = UW/D_a_, the microchannel length-to-width ratio L_N_ = L/W, the normalized diffusion coefficient D_N_ = D_b_/D_a_, and the normalized solvent flow rate Q_N_ = Q_solvent_/(∑Q_i_). For equal flow rates of the reagents A and B, the resulting scaling law was:

(2) $${\mathrm{Pe}}_{\mathrm{in}}\approx \frac{8}{3}L_{N}{\left(\frac{1+\sqrt{D_{N}}}{Q_{N}}\right)}^{2}$$

and the reaction front location:

(3) $$y_{r}=\frac{W}{2}-\frac{W}{2}Q_{N}\frac{\sqrt{D_{N}}-1}{\sqrt{D_{N}}+1}$$

Equation (3) allows the “turning on” and “turning off” of the reaction in the main channel on demand for arbitrary microchannel geometries (L_N_) and reaction pairs (D_N_) by varying Pe and Q_N_ numbers. Equation (4) predicts the reaction front location across the main channel and, thus, the presence of the reaction product in a specific output.

A characteristic feature of reacting colloid and polymer systems is a combination of their low diffusivities and relatively high rates of surfactant binding by polymer [50], due to electrostatic interactions and the hydrophobic effect. To evaluate competing kinetic and diffusion factors, we calculated the Damköhler number, that is the ratio of reaction and diffusion rates: Da = ${\mathrm{kC}}^{0}W^{2}/D_{a}$, where C^0^ is the initial polymer concentration. We used an approximate value of the rate constant that was set to 10^4^. Such a value is close to the reported rates of polymer-surfactant interactions [49,50]. For the PDADMAC-SDS reaction pair and 200–300 μm wide microchannels, Da ≈ 10^4^–10^5^, the reagents should interact almost instantaneously when they meet by diffusion. We could consider that the rate of reaction was not a governing factor in the process kinetics. This agreed with papers reporting that diffusion-controlled reactions in microchannels depend weakly on reaction rate constants [18].

For the fast diffusion-limited complexation processes studied in this paper, we could assume, therefore, that the reaction completed when the reagents mixed in the main channel by diffusion. For the geometry in Figure 2, the mixing time [53] was defined as follows:

(4) $$t_{\mathrm{mix}}=\frac{{\left(W-y_{r}\right)}^{2}}{D_{a}}=\frac{y_{r}{}^{2}}{D_{b}}\approx \frac{L}{3/2U}$$

The respective scaling law for chemical equilibrium was:

(5) $${\mathrm{Pe}}_{\mathrm{eq}}\approx \frac{2}{3}L_{N}{\left(1+\sqrt{D_{N}}\right)}^{2}$$

A more detailed derivation of Equations (2)–(5) is provided in Appendix A.

By setting the flow conditions close to those predicted by Equation (5), we could achieve product accumulation across the main channel end and its release from multiple, or all, outputs.

The Peclet number is a widely used microfluidic similarity criterion, which characterizes the ratio of convection and diffusion rates in a microchannel [53,54]. In Equations (2) and (5), the Peclet number depends on other dimensionless numbers that combine microchannel length and width (L_N_), diffusivities of reagents (D_N_) and a flow rate pattern (Q_N_). Therefore, these equations allowed evaluation of the microchip operation conditions that corresponded to reaction start and completion for arbitrary reaction pairs, microchip geometries, and operation modes.

### 3.3. Numerical and Experimental Verification of the Scaling Laws

The dimensionless numbers, Pe_in_ and Pe_eq_, are expected to provide only an evaluation of reaction conditions in a microchip. Firstly, a characteristic diffusion time does not state the exact concentration of diffusing molecules at a specific distance from the flow of a reagent. Secondly, detection of a chemical signal at microchip outputs depends on the sensitivity of an applicable analytical method.

The first step to verify the scaling laws was to perform the numerical simulation of the processes, shown in Figure 2, by solving Equation (1) with Matlab, and comparing the results with Pe_in_ and Pe_eq_ predictions.

The mathematical model suitable for numerical simulations should consider convection, diffusion, and kinetic processes in microfluidic flows. Fundamentally, it is necessary to analyze continuity and the Navier-Stokes equations. In the experiments, however, the inlet flows of the reagents and the solvent were stationary. The viscosity of polymer and surfactant solutions was close to that of a pure solvent. The flows in microchannels were laminar (the calculated Reynold number was Re < 5 for all the experiments). Such conditions are standard for single-phase microfluidic reactive flows [53]. We could, therefore, use the solution of the Navier-Stokes equations, which predicts a parabolic flow velocity field in a microchannel, that is a standard approach in literature to model such microfluidic systems [49,53,54,55,56]. For a rectangular microchannel and the coordinates shown in Figure 2, the velocity field could be approximated by the following equation:

(6) $$U\left(y\right)=\frac{3}{2}U\left(1-{\left(1-\frac{y}{W}\right)}^{2}\right)$$

In the main channel (Figure 2), therefore, there was a pressure-driven laminar flow of solutions along the X axis (the axial convection of the reacting species). The reagents also diffused across the main channel from the side flows to the reaction site in the solvent flow. In stationary flow conditions, we could neglect the axial diffusion of the reacting species. The equation that described the concentration distribution of the reagent A in the main channel included the axial convection component $A_{t}^{\prime }=U\left(y\right)A_{x}^{\prime }$ and the radial diffusion component (Fick’s law): $A_{t}^{\prime }=D_{a}A_{y}^{\prime \prime }$, where A is the molar concentration of surfactant, $A_{t}^{\prime }$ and $A_{x}^{\prime }$ are its first order partial derivatives by time and the X axis, respectively, $A_{y}^{\prime \prime }$ is its second-order partial derivative by the Y axis, and $D_{a}$ is the diffusion coefficient of the reagent A. In the stationary inlet flow conditions of the performed microfluidic experiments ($A_{t}^{\prime }=0)$, we obtained the convection-diffusion equation [53]:

(7) $$U\left(y\right)A_{x}^{\prime }=D_{a}A_{y}^{\prime \prime }$$

We should also consider the reaction occurring between the reagents A and B. Surfactant binding by polymer can be described by a second-order reaction A + B = C, where B is the molar concentration of polymer binding cites (for PDADMAC, it is equal to the molar concentration of its monomer units) and C is the concentration of polymer binding centers with bound surfactant ions. The rate law for this reaction is ${\left[A\right]}_{t}^{\prime }=-\mathrm{kAB}$, where k is the rate constant. This kinetic rate law contributed to Equation (7) as the sink term. In the stationary conditions of the microfluidic experiments, the resulting equation predicted the concentration distribution of reagent A in the main channel [53]:

(8) $$U\left(y\right)A_{x}^{\prime }=D_{a}A_{y}^{\prime \prime }-\mathrm{kAB}$$

Equation (8) combines the convection term with a parabolic velocity field, the diffusion term introduced according to Fick’s law, and the kinetic term representing the chemical reaction of polyelectrolyte-surfactant complexation.

The same equations were derived for the polymer B and the product C. The resulting system represented the complete set of convection-diffusion reaction equations [53] that described the concentration distribution of the reaction species in the main channel:

(9) $$\left\{\begin{array}{c} U\left(y\right)A_{x}^{\prime }=D_{a}A_{y}^{\prime \prime }-kAB\\ U\left(y\right)B_{x}^{\prime }=D_{b}B_{y}^{\prime \prime }-kAB\\ U\left(y\right)C_{x}^{\prime }=D_{c}C_{y}^{\prime \prime }+kAB \end{array}\right.$$

where U(y) is the flow velocity; D_a_, D_b_, and D_c_ are the diffusivities of surfactant, polymer and the reaction product.

A more detailed derivation of the Equation (9) is provided in Supplementary Materials and Figure S1.

To complete the mathematical model, we needed to introduce the boundary conditions for the main channel. At the junction point (x = 0), the concentration of a reagent was equal to its initial concentration at the inlet flow and was zero elsewhere. The concentration of the product was zero at the junction point. If y_a_ and y_b_ were the boundaries of the A and B inlet flows (Figure 2), then we could introduce the following Dirichlet boundary conditions for x = 0:

(10) $$\left\{\begin{array}{c} \left[A\right]\left(x=0,y\right)=\left\{\begin{array}{c} {\left[A\right]}^{0},y\geq y_{a}\\ 0,y<y_{a} \end{array}\right.\\ \left[B\right]\left(x=0,y\right)=\left\{\begin{array}{c} {\left[B\right]}^{0},y\leq y_{b}\\ 0,y>y_{b} \end{array}\right.\\ \left[C\right]\left(x=0,y\right)=0\;\;\;\;\;\;\;\;\;\;\;\;\;\;\;\;\;\;\;\;\; \end{array}\right.$$

The coordinates y_a_ and y_b_ depend on surfactant, polymer, and solvent flow widths. In turn, these flow widths are proportional to the surfactant, polymer, and solvent flow rates in identical conditions of their flow paths to the main channel. If Q_a_, Q_b_, and Q_s_ are the polymer, surfactant, and solvent flow rates, respectively, then (for the geometry in Figure 2):

(11) $$y_{a}=\frac{Q_{b}+{\;Q}_{s}}{\sum Q}W,{\;y}_{b}=\frac{Q_{b}}{\sum Q}W$$

With the addition of Equation (11) to the boundary conditions represented by Equation (10), we could cover all possible cases of polymer, surfactant, and solvent flow widths in the main channel by setting them with the respective flow rates.

We also needed to introduce the boundary conditions for the main channel walls (y = 0 and y = W in Figure 2). They were derived from the assumption that the reaction species did not penetrate through microchannel walls, so resulting in a zero-radial flux at the walls [53]. For the reagent A, we could formalize it as the following Neumann boundary conditions [53]:

(12) $$\left\{\begin{array}{c} {\left[A\right]}_{y}^{\prime }\left(y=0\right)=0\\ {\left[A\right]}_{y}^{\prime }\left(y=W\right)=0 \end{array}\right.$$

The same boundary conditions were introduced for the reagent B and the product C.

Equations (9)–(12) represent a mathematical model suitable for numerical simulations to predict the concentration profiles of the reacting species in the main channel. These equations were further transformed into a dimensionless form to be solved in Matlab (2021a, MathWorks, Inc., Natick, MA, USA). More details of the dimensional analysis are provided in the Supplementary Materials and Figure S1.

In the numerical simulations, we used the following diffusion coefficients of the reagents: D_PDADMAC_ ≈ 50 μm^2^/s found by DLS and D_SDS_ ≈ 500 μm^2^/s [51].

**Figure 3:**
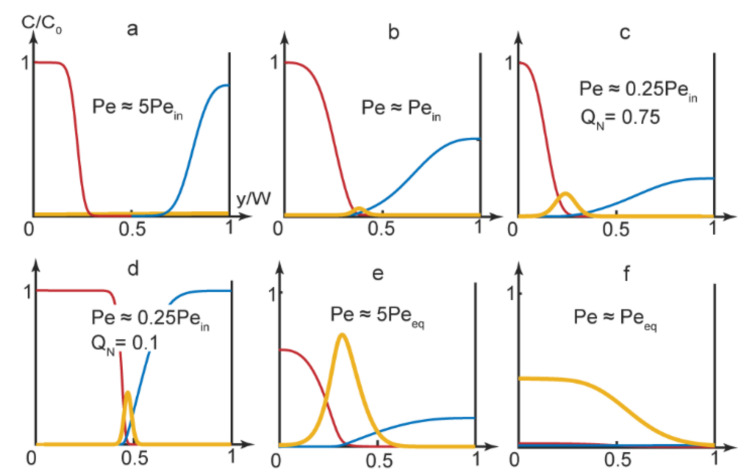
Numerical simulations results at the main channel end (x = L) for the PDADMAC-SDS complexation reaction. Concentration curves: blue—PDADMAC, red—SDS, yellow—complex. Flow rates: (**a**) A, B—5 μL/min, solvent—10 μL/min; (**b**) A, B—5 μL/min, solvent—3 μL/min; (**c**) A, B—0.2 μL/min, solvent—1.2 μL/min; (**d**) A, B—10 μL/min, solvent—2 μL/min; (**e**) A, B, solvent—1 μL/min; (**f**) A, B, solvent—0.1 μL/min. The main channel width = 300 µm and length = 15 mm.

Equation (9) was solved in Matlab with the boundary conditions represented by Equations (10) and (12). The flowrates of the reagents were selected to provide conditions below, above or equal to the respective threshold Peclet numbers. Figure 3 demonstrates the results of the numerical simulations for the main channel end, where the reactive species distributed between 3 outputs.

As we can see in Figure 3, the reaction product could accumulate in various parts of the main channel, depending on the experimental conditions. It could, therefore, release through various single or multiple outputs.

In Figure 3a’s simulation, the product did not form in the center of the main channel and the reagents left the microchip through the side outputs. Figure 3b demonstrates an approximation of the start of the complexation reaction (turning the chip “On”). The product was supposed to release through the central output

By using Equation (4), we also evaluated conditions when the reaction front displaced towards another output. With a broad solvent flow (Q_N_→1), the reaction front displaced to the left part of the microchannel (y_r_ ≈ 0.25–0.35). Predictions of Equation (4) were verified by numerical modeling of the PDADMAC-SDS reaction pair. The results of simulations (Figure 3c) agreed with Equation (4)’s evaluations. Under Q_N_→1 conditions, the reaction product mostly accumulated near the side of the main channel and was supposed to release through the side output.

Additional numerical simulations were performed for Q_N_→1 conditions for reaction pairs with various diffusivities (D_N_ = 1–10). The results showed that for the reagents with similar diffusivities (D_N_ ≈ 1), the reaction product emerged in the center of the microchannel at all applicable flow rates. For reagents with different diffusivities, numerical simulations showed that the reaction front displaced towards the flow of a reagent with a lower diffusion coefficient. In our case, the reaction occurred near the flow of slowly diffusing polymer macromolecules. Therefore, by varying solvent flow widths for reagents with different diffusivities, we could switch the reaction product released between different outputs.

By minimizing the solvent flow rate, we eliminated the gap between the reacting flows. The product formed in the center of the main channel and was supposed to release through the central output (Figure 3d).

Finally, by selecting microchip operation modes that corresponded to lower Peclet numbers, approaching equilibrium values, we synthesized polymer-surfactant complexes that occupied two thirds of the main channel first (Figure 3e) and, then, the entire main channel (Figure 3f). In such conditions, we could achieve the release of the reaction product through multiple or all outputs.

Additional details on the numerical simulation results for the entire main channel, which agreed with Figure 3, are provided in the Figure S2 in the Supplementary Materials.

**Figure 4:**
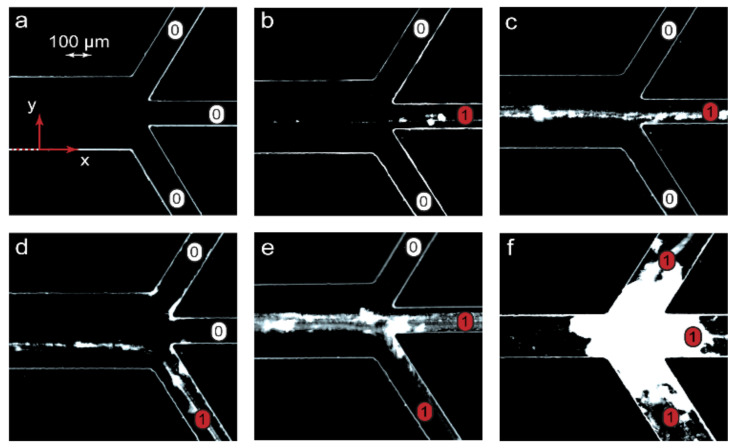
Visualization of chemical signals near microchip outputs (x = L) in the microfluidic experiments with the PDADMAC-SDS reaction pair: “1”—the product is present in an output (precipitate), “0”—no product in an output (no precipitate). The flow rates of the reagents and the solvent were identical to those of the numerical simulations shown in Figure 3: (**a**) A, B—5 μL/min, solvent—10 μL/min; (**b**) A, B—5 μL/min, solvent—3 μL/min; (**c**) A, B—0.2 μL/min, solvent—1.2 μL/min; (**d**) A, B—10 μL/min, solvent—2 μL/min; (**e**) A, B, solvent—1 μL/min; (**f**) A, B, solvent—0.1 μL/min. The main channel width = 300 µm and length = 15 mm.

The second step to verifying the scaling laws was to perform microfluidic experiments in conditions matching those used for the numerical simulations. A peculiar feature of reacting polymer-surfactant systems in a nonequilibrium microfluidic confinement is that their complexes precipitate slightly on the bottom of a microchannel along the reaction front. The respective equilibrium macroscopic solutions, however, remained homogeneous. The reason for such an effect might be a preferable binding of surfactant ions by macromolecules available at the reaction front and the resulting reduction in solubility of nonequilibrium polymer-surfactant aggregates in the center of the main channel. Precipitation allowed an easy on-chip tracking of the reaction front locations by optical microscopy and provided a simplified visual detection of the resulting chemical signals at the chip outputs in addition to DLS. Figure 4 demonstrates the processed negative optical microscopy images of microchip outputs with precipitates marking the reaction product pathways.

Figure 4 visualizes the emergence of a chemical signal (the reaction product) and its release through various microchip outputs. The images in Figure 4 agreed with the results of numerical simulations represented in Figure 3. At Pe > Pe_in_, the microchannel was clean (Figure 4a), indicating that no complexation reaction occurred between PDADMAC and SDS. The chip was, therefore, in the “off” state. Traces of precipitated PDADMAC-SDS aggregates appeared at conditions that corresponded to the start of the complexation reaction (Pe = Pe_in_). At Pe < Pe_in_, a chemical signal marked by partly precipitated complexes migrated between microchip outputs if different solvent flow rates were set (Figure 4c,d). Finally, the precipitates were observed at multiple outputs when the reaction system approached equilibrium (Figure 4e,f).

At low flow rates, precipitation might lead to microchip clogging (Figure 4f) and failure. A series of additional microfluidic experiments performed for the PDADMAC-SDS pair, revealed that precipitation was reversible and could easily be eliminated or minimized by performing the complexation reaction in 20–30 wt.% ethanol, which was more tolerant to hydrophobic colloidal particles. For applications of such microfluidic devices, we should, therefore, select either an appropriate solvent or polymer-surfactant reaction pairs that do not tend to precipitate at the respective ratios of their concentrations.

We performed activation of a macromolecular chemical signal and switched it between multiple outputs by just varying the flow rates According to Equations (4) and (6), we could achieve conditions shown in Figure 3 with other combinations of flow rates and also other microchannel lengths, widths and reaction pairs. Additional selected numerical simulations were performed for microchannel widths of 100–300 μm, lengths of 15–300 mm, flow rates of 0.2–100 μL/min and reaction pairs with diffusivities of 10–1000 μm^2^/s. Equations (4) and (6) provided a satisfactory agreement with the results of simulations. These simulations were selectively verified for the PDADMAC-SDS reaction pair in microchips with different geometries (200 μm widths and 30–100 mm lengths). The experiments also agreed with numerical simulations and demonstrated the product output states were similar to those shown in Figure 4.

### 3.4. Detecting Chemical Signals and Achieving Logic Gate States

For detecting supramolecular chemical signals, microfluidic samples of PDADMAC-SDS solutions were collected at chip outputs and analyzed by DLS. The results are summarized in Table 1. The samples were taken from the microchips in the same experimental conditions as demonstrated in Figure 4.

The DLS data of the samples collected at the microchip outputs agreed with the numerical simulations represented by Figure 3 and the visual results shown in Figure 4. Before the start of the reaction (conditions “a”), particles of about 10 nm in diameter were detected in the sample taken at output “3”. The size of these particles corresponded to those of the initial PDADMAC macromolecules, which were supposed to release through output “3”.

The samples released through outputs “1” and “2” in the experimental conditions “a” were also analyzed by dynamic light scattering. In output “1”, DLS detected only particles of approximately 2–3 nm in diameter. These particles were supposed to be releasing surfactant molecules, which were fed through the input “A”. Similar results were obtained for the output 2, that might indicate that traces of surfactant penetrated to the output 2 flow by diffusion.

The size range of 2–3 nm is, however, close to the lower DLS detection threshold and high-precision measurements require additional preparation of samples that was not necessary in this work, as the chemical signal we intended to detect was in a far larger size range of ~100 nm. Therefore, we approximated the results as “<10 nm” in Table 1 for the conditions “a” and further experiments.

Under the conditions, “b”, particles of a broad size range (10–100 nm) were detected in the central output. They might have been both individual polymer macromolecules and aggregated complexes. Such a chemical signal was unstable and possibly indicated a large detection error due to a very low concentration of the reaction product in the Pe = Pe_in_ conditions. Similar unstable results were obtained by performing the complexation reaction in conditions close to Pe_in_: 0.5Pe_in_ < Pe < 2Pe_in_.

Under the conditions, “c”, with a broad solvent flow, the size of particles in the lower output was about 70–80 nm, which was above the selected signal detection threshold (50 nm). A smaller size of particles agreed with the size of complexes synthesized in macroscopic conditions in similar polymer-surfactant solutions diluted by large solvent additives. The readout of the central output was unstable and varied in each measurement, indicating that certain macromolecules or product aggregates might have released through the central output. Setting higher Q_N_ values allowed us to shift the reaction front to the lower output of the chip and improve the readability of the signal.

Under the conditions, “d”, Pe ≈ 0.25Pe_in_, the product concentration in the central output was relatively high, according to the numerical simulations, and provided a stable chemical signal, represented by 100 nm particles.

Under the conditions, “f” and “e”, Pe→Pe_eq_ and polymer-surfactant aggregates of 100–150 nm in diameter were observed in multiple, and all, the outputs, respectively.

The digital equivalents of the reaction product absence and presence in the output channels were 0 and 1, respectively. Three outputs, therefore, provided a rich variety of digital chemical signal readouts set by the dimensionless numbers in the scaling laws (Equations (3), (4) and (6)).

To evaluate a possible impact of PDADMAC and SDS concentrations on chemical signal generation and detection, numerical modeling and experiments were also performed with concentrations of an order of lower magnitude (6.1 × 10^−4^ mol/L). At these concentrations, numerical simulations provided results similar to those in Figure 3. Accumulation of precipitates in the experiments was much lower than that shown in Figure 4 for higher concentrations of reacting species. The signal detection was unstable under the conditions “b” and “d” but provided reliable results in other conditions.

Selected experiments were also performed in conditions “b”, “c”, and “d” at concentrations of the reagents above those corresponding to the critical micellization concentration (CMC) of SDS (8.4 × 10^−3^ mol/L). At concentrations above CMC, surfactant aggregates with diffusivities different from those of individual surfactant molecules appeared in the solutions. In such conditions, optical microscopy images showed that the precipitate lines marking the reaction front were displaced by 20–30 μm from their original locations. Consideration of micelles requires, therefore, a different mathematical model.

Table 2 summarizes the digital readouts of the microfluidic chips with respect to the scaling laws. Table 1, and Figure 3 and Figure 4 also show the corresponding logic gate states of the chips.

The “a” state was, therefore, a dynamic “off” state and no product emerged in the microchip, although the reagents continued to flow and release through the side outputs. The “b” state was the microchip activation threshold. In this state, a reliable product generation was unstable. In the states from “c” to “f”, the chip performed as a chemical signal switch between various microchip outputs.

The microchip operational modes, summarized in Table 2, provide various logic gate states. In the “off” mode, the logic states were AND = 0 and OR = 0. In the activation and switching modes, AND = 0 and OR = 1 until the outputs were fully occupied by the reaction product (AND = 1, OR = 1).

Thus, the threshold Pe numbers allowed evaluation of the reaction initiation and completion conditions in microchips. For a stable, reproducible, and detectable generation of chemical signals, we should select microchip operation conditions that exceed, or fall behind, the threshold Pe_in_ values.

We could implement a two-step strategy of chemical signal activation and switching for a variety of reaction pairs in microfluidic devices. Firstly, we evaluated the reaction front initiation and switching conditions with the threshold dimensionless numbers Pe_in_ and Pe_eq_ and the reaction front location y_r_. Secondly, we verified the results with the numerical script and obtained more precise microchip operation parameters for generating a stable chemical signal. Chemical signal readouts and logic gate states could be changed dynamically by programming microfluidic pumps.

## 4. Discussion

Laboratory-on-chip microdevices with built-in chemical information processing capabilities offer additional options for integrating microfluidics with computing systems. Biomedical applications of laboratory-on-chip, such as molecular diagnostics, synthesis of drug delivery nanocarriers, and prototyping organs on chip, are among the most vibrant trends in science and technology.

The approach to generating chemical signals and logic gates demonstrated in this work allows the design of components of microfluidic circuits for the above applications.

Firstly, detecting surfactants, micelles, polymer macromolecules, or their aggregates, at specific outputs of microfluidic logic gate chips allows important conclusions to be made on the composition of an analyte and the properties of its components. Logic gates and scaling laws allow integration of the detection results with software algorithms, which can facilitate and speed-up the diagnostic process. Logic gate states can also provide convenient feedback on the properties of polymer-surfactant nanoparticles to optimize microchip operation parameters for.

Secondly, the designed circuit can be installed into microfluidic devices, which contain immobilized cells or enzymes. By initiating chemical signals and switching them between multiple outputs, we can provide an on-demand exposure of certain biological objects to polymer-surfactant systems with drug carrying capabilities in cascade microfluidic systems. It allows the testing of drug delivery systems in variable conditions that are close to in-vivo.

Thirdly, the proposed microfluidic circuit can be installed into organ-on-chip prototypes to selectively route chemical signals to certain cell cultures or collect cell responses and analyze the resulting logic gate states to characterize metabolic processes, according to pre-designed software algorithms.

The merit of the proposed approach over other known microfluidic techniques for chemical signal and logic gate generation is that it does not require microdroplets, complex microfluidic designs, or additional tools. This method utilizes reaction fronts emerging in microchannels that release selectively through specific output as discrete chemical signals.

In this work, however, we have not developed a finalized microfluidic device. We designed and tested a microfluidic circuit, which can be installed into laboratory-on-chip or organ-on-chip prototypes as a chemical signal generating component. The focus of the research was to formalize the operation conditions of this circuit as the scaling laws, which can be conveniently integrated into software algorithms that control the operation of microfluidic devices.

The detection of chemical signals by dynamic light scattering is appropriate for laboratory-scale experiments. If detection and visualization of chemical signals is required in final microfluidic devices, a faster on-chip detection method should be considered, such as optofluidic or electrochemical tools, or fluorescence microscopy. Integration of such methods into the proposed microfluidic circuits is among our future research highlights.

We considered only a limited number of factors which can be used to control supramolecular chemical signal generation in microchips. We varied just the flow rates of the reagents and the solvent, which was sufficient to generate chemical signals and different logic gates. Our subsequent research activities will focus on varying the main channel geometry (lengths and widths) in a broad range and the use of additional polymer and colloid reaction pairs with various diffusivities and concentrations in different solvents and at various pH values. This will allow the testing of emerging chemical signal responses and logic gate states suitable for specific biomedical applications of microfluidic devices.

## 5. Conclusions

We developed a microfluidic circuit design, numerical model, and operating modes that allow the activation of a chemical signal in polyelectrolyte-surfactant reactive flows and selective switching between multiple outputs. The model and its experimental verification revealed a variety of factors that facilitate the control of a chemical signal response of a microchip: diffusivities of the reagents, microchip geometry, and the input flow rate pattern. We combined these factors into scaling laws that allow the evaluation of the reaction initiation and completion and the reaction front location in microchips. In combination with numerical simulations, these scaling laws provide microfluidic chips with chemical logic gate capabilities.

The proposed microfluidic reaction circuits could be suitable for on-demand exposure of biological objects to supramolecular chemical signals in organ-on-chip prototypes, generation of control commands in laboratory-on-chip instruments, or the introduction of logic gates into cascade microfluidic chips.

## Figures and Tables

**Table 1:** Detection of chemical signals by dynamic light scattering.

Conditions	a	b	c	d	e	f
Scaling Law	Pe ≥ Pe_in_		Pe < Pe_eq_		Pe → Pe_eq_	
Hydrodynamic diameter, nm						
Output 1	<10	<10	<10	<10	<10	150
Output 2	<10	10–100	100	10–70	120	150
Output 3	10	10	10	70	100	150

**Table 2:** Digital readouts and microfluidic chip states at various values of Peclet number.

Conditions		a	b	c	d	e	f
Scaling Law		Pe ≥ Pe_in_		Pe < Pe_eq_		Pe → Pe_eq_	
Microfluidic device mode		Off	On, unstable	On Signal switching			
Readout (reaction product yes/no)	Output 1	⓪	⓪	⓪	⓪	⓪	❶
	Output 2	⓪	❶	❶	⓪	❶	❶
	Output 3	⓪	⓪	⓪	❶	❶	❶
Logic gate	AND	0	0				1
	OR	0	1				1

## Supplementary Materials

The following supporting information can be downloaded at: https://www.mdpi.com/article/10.3390/mi13101778/s1, Figure S1: Geometry of a microfluidic chip with the length L and the width W. Figure S2: Numerical simulations results at the main channel (x = L, y = W) for the PDADMAC-SDS complex concentration field. Flow rates: (a) A, B—5 μL/min, solvent—10 μL/min; (b) A, B—5 μL/min, solvent—3 μL/min; (c) A, B—0.2 μL/min, solvent—1.2 μL/min; (d) A, B—10 μL/min, solvent—2 μL/min; (e) A, B, solvent—1 μL/min; (f) A, B, solvent—0.1 μL/min. The main channel width = 300 µm and length = 15 mm.

## Appendix A

Determination of Scaling Laws for Reaction Initiation and Completion in a Microfluidic Channel.

Step 1. Determine the reaction front y_r_ location in the microchannel.

The starting point is the equation that estimates the diffusion time of the reagents to the site of reaction, as shown in Figure 2. As the reaction starts and proceeds in the central part of the microchannel, we can approximate the flow velocity in the reaction front zone as the maximum flow velocity in the microchannel: U_max_ = 3/2U, where U is the average flow velocity calculated from the microchannel width W, height H, and the total flow rate Q: U = Q/(WH):

(A1) $$t_{\mathrm{in}}=\frac{{\left(y_{a}-y_{r}\right)}^{2}}{4D_{a}}=\frac{{\left(y_{r}-y_{b}\right)}^{2}}{4D_{b}}=\frac{L}{3/2U}$$

Consider the following segment of this equation:

(A2) $$\frac{{\left(y_{a}-y_{r}\right)}^{2}}{4D_{a}}=\frac{{\left(y_{r}-y_{b}\right)}^{2}}{4D_{b}}$$

and transform it:

(A3) $$\frac{y_{r}-y_{b}}{y_{a}-y_{r}}=\sqrt{\frac{D_{b}}{D_{a}}}$$

Introduce the dimensionless similarity criterion D_N_:

(A4) $$D_{N}=\sqrt{\frac{D_{b}}{D_{a}}}$$

then:

(A5) $$\frac{y_{r}-y_{b}}{y_{a}-y_{r}}=\sqrt{D_{N}}$$

Continue transformations:

(A6) $$y_{r}-y_{b}=\sqrt{D_{N}}\left(y_{a}-y_{r}\right)$$

then:

(A7) $$y_{r}+\sqrt{D_{N}}y_{r}=\sqrt{D_{N}}y_{a}+y_{b}$$

Introduce the flow rates:

Q_a_ is the flow rate of the reagent A, Q_b_ is the flow rate of the reagent A, and Q_S_ is the flow rate of the solvent.

Calculate the width of the solvent flow from the flow rates:

(A8) $$y_{a}-y_{b}=W\left(\frac{Q_{s}}{Q_{a}+Q_{b}+Q_{s}}\right)$$

Introduce the normalized flow rate Q_N_ that is equal to the fraction of the microchannel width occupied by the solvent flow:

(A9) $$Q_{N}=\frac{Q_{s}}{Q_{a}+Q_{b}+Q_{s}}$$

Set the flow rates of the reagents Q_a_ = Q_b_ in all the experiments. In such conditions, reagent A and B flows always have the same width. This width is set by the normalized solvent flow rate Q_N_. In the coordinates shown in Figure 1, the flow widths of the reagents A and B are (W−Q_a_) and (y_b_−0), respectively. If these flow rates are equal in all the experiments, then:

(A10) $$\left(W-y_{a}\right)=y_{b}=W\frac{1-Q_{N}}{2}$$

The y_a_ coordinate in Figure 1:

(A11) $$y_{a}=W-W\frac{1-Q_{N}}{2}=W\frac{1+Q_{N}}{2}$$

Substitute y_a_ and y_b_ in Equation (A7) according to Equations (A10) and (A11):

(A12) $$y_{r}+\sqrt{D_{N}}y_{r}=\sqrt{D_{N}}W\left(\frac{1+Q_{N}}{2}\right)+W\frac{1-Q_{N}}{2}$$

Transform Equation (A12):

(A13) $$y_{r}\left(1+\sqrt{D_{N}}\right)=\frac{\sqrt{D_{N}}W+\sqrt{D_{N}}{\mathrm{WQ}}_{N}+W-{\mathrm{WQ}}_{N}}{2}$$

then:

(A14) $$y_{r}\left(1+\sqrt{D_{N}}\right)=\frac{W\left(1+\sqrt{D_{N}}\right)-{\mathrm{WQ}}_{N}\left(1-\sqrt{D_{N}}\right)}{2}$$

Dividing Equation (A14) by $\left(1+\sqrt{D_{N}}\right)$, we finally get the equation that predicts the radial position of the reaction front:

(A15) $$y_{r}=\frac{W}{2}-\frac{W}{2}Q_{N}\frac{\left(1-\sqrt{D_{N}}\right)}{\left(1+\sqrt{D_{N}}\right)}$$

The radial coordinate of the reaction front at the main channel end allows prediction of the release of the reaction product from the specific output of the microfluidic chip after the start of reaction.

Step 2. Derive the scaling law for the reaction initiation at the main channel output.

We proceed with analyzing the following segment of Equation (A1):

(A16) $$\frac{{\left(y_{a}-y_{r}\right)}^{2}}{4D_{a}}\approx \frac{L}{3/2U}$$

Transform it by making substitutions according to Equations (A11) and (A15):

(A17) $${\left(W\frac{1+Q_{N}}{2}-\frac{W}{2}+\frac{W}{2}Q_{N}\frac{\left(1-\sqrt{D_{N}}\right)}{\left(1+\sqrt{D_{N}}\right)}\right)}^{2}\approx 4D_{a}\frac{L}{3/2U}$$

Divide by W^2^:

(A18) $${\left(\frac{1+Q_{N}}{2}-\frac{1}{2}+\frac{1}{2}Q_{N}\frac{\left(1-\sqrt{D_{N}}\right)}{\left(1+\sqrt{D_{N}}\right)}\right)}^{2}\approx 4\frac{D_{a}}{3/2\mathrm{UW}}\frac{L}{W}$$

Transform the left part of Equation (A18):

(A19) $${\left(\frac{1}{2}+\frac{Q_{N}}{2}-\frac{1}{2}+\frac{1}{2}Q_{N}\frac{\left(1-\sqrt{D_{N}}\right)}{\left(1+\sqrt{D_{N}}\right)}\right)}^{2}\approx \frac{8}{3}\frac{D_{a}}{\mathrm{UW}}\frac{L}{W}$$

then:

(A20) $${\left(\frac{Q_{N}}{2}+\frac{Q_{N}}{2}\frac{\left(1-\sqrt{D_{N}}\right)}{\left(1+\sqrt{D_{N}}\right)}\right)}^{2}\approx \frac{8}{3}\frac{D_{a}}{\mathrm{UW}}\frac{L}{W}$$

Transform Equation (A20):

(A21) $${\left(\frac{Q_{N}\left(1+\sqrt{D_{N}}\right)+Q_{N}\left(1-\sqrt{D_{N}}\right)}{2\left(1+\sqrt{D_{N}}\right)}\right)}^{2}\approx \frac{8}{3}\frac{D_{a}}{\mathrm{UW}}\frac{L}{W}$$

to get:

(A22) $${\left(\frac{Q_{N}+Q_{N}\sqrt{D_{N}}+Q_{N}-Q_{N}\sqrt{D_{N}}}{2\left(1+\sqrt{D_{N}}\right)}\right)}^{2}\approx \frac{8}{3}\frac{D_{a}}{\mathrm{UW}}\frac{L}{W}$$

Finally:

(A23) $${\left(\frac{Q_{N}}{1+\sqrt{D_{N}}}\right)}^{2}\approx \frac{8}{3}\frac{D_{a}}{\mathrm{UW}}\frac{L}{W}$$

As UW/D_a_ = Pe (Peclet number), and L/W = L_N_ (the microchannel length-to-width ratio) then:

(A24) $${\left(\frac{Q_{N}}{1+\sqrt{D_{N}}}\right)}^{2}\approx \frac{8}{3}\frac{L_{N}}{\mathrm{Pe}}$$

The Peclet number that corresponds to reaction initiation at the main channel output (the distance L from the junction point):

(A25) $${\mathrm{Pe}}_{\mathrm{in}}\approx \frac{8}{3}L_{N}{\left(\frac{1+\sqrt{D_{N}}}{Q_{N}}\right)}^{2}$$

This Peclet number Pe_in_ depends on the following similarity criteria: L_N_ = L/W (the microchannel length-to-width ratio), $D_{N}=\sqrt{\frac{D_{b}}{D_{a}}}$ (the ratio of diffusivities of reacting species), and QN = Q_S_/ΣQ (the width of the central solvent flow).

By setting the Peclet number above Pe_in_, we set the microchip to the “off” state and the reaction does not start at the main channel output.

By setting the Peclet number equal to Pe_in_, we set the microchip to the “on” state and the reaction starts at the main channel output. The product releases from the specific microchip output predicted by Equation (A15).

Step 3. Derive the scaling law for the reaction completion at the main channel output.

Assume that the reagents A and B react almost instantaneously at the site of reaction, so the reaction completes when these reagents mix. In such conditions, the reaction front y_r_ creates two separate zones in the main channel with only A and B molecules diffusing to the site of reaction: W−y_r_ and y_r_−0, respectively. We can, therefore, define the mixing time as follows:

(A26) $$t_{\mathrm{mix}}=\frac{{\left(W-y_{r}\right)}^{2}}{D_{a}}=\frac{y_{r}{}^{2}}{D_{b}}\approx \frac{L}{3/2U}$$

Consider the following segment of Equation (A23):

(A27) $$\frac{{\left(W-y_{r}\right)}^{2}}{D_{a}}=\frac{y_{r}{}^{2}}{D_{b}}$$

and transform it as we did for Equations (A1) and (A2):

(A28) $$\frac{y_{r}}{W-y_{r}}=\sqrt{D_{N}}$$

then:

(A29) $$y_{r}+{\;y}_{r}\sqrt{D_{N}}=W\sqrt{D_{N}}$$

and:

(A30) $$y_{r}=W\frac{\sqrt{D_{N}}}{1+\sqrt{D_{N}}}$$

Consider the following segment of Equation (A26):

(A31) $$\frac{{\left(W-y_{r}\right)}^{2}}{D_{a}}\approx \frac{L}{3/2U}$$

Introduce a substitution for y_r_, according to Equation (A31):

(A32) $${\left(W-W\frac{\sqrt{D_{N}}}{1+\sqrt{D_{N}}}\right)}^{2}\approx \frac{{\mathrm{LD}}_{a}}{3/2U}$$

Divide by W^2^:

(A33) $${\left(1-\frac{\sqrt{D_{N}}}{1+\sqrt{D_{N}}}\right)}^{2}\approx \frac{D_{a}}{3/2\mathrm{UW}}\frac{L}{W}$$

Simplify the left part of Equation (A33):

(A34) $${\left(\frac{1}{1+\sqrt{D_{N}}}\right)}^{2}\approx \frac{D_{a}}{3/2\mathrm{UW}}\frac{L}{W}$$

As UW/D_a_ = Pe (Peclet number), and L/W = L_N_ (the microchannel length-to-width ratio) then:

(A35) $${\left(\frac{1}{1+\sqrt{D_{N}}}\right)}^{2}\approx \frac{L_{N}}{3/2\mathrm{Pe}}$$

Thus, the Peclet number that corresponds to reaction completion at the main channel output (chemical equilibrium) is:

(A36) $${\mathrm{Pe}}_{\mathrm{eq}}\approx \frac{2}{3}L_{N}{\left(1+\sqrt{D_{N}}\right)}^{2}$$

By setting the microchip operation parameters close to Pe_eq_, we can achieve considerable product accumulation at the main channel end and, thus, its release from multiple, or all, the outputs.

By selecting the microchip operation parameters between Pe_in_ and Pe_eq_, according to Equations (A15) and (A34), we can achieve product release from various single or multiple outputs.
