# Supplementary material for: Activation and Switching of Supramolecular Chemical Signals in Multi-Output Microfluidic Devices
Source: Micromachines (Basel). 2022 Oct 19;13(10):1778. doi: 10.3390/mi13101778 (PMC9611873; doi:10.3390/mi13101778)
Supplement: Supplementary file 1 [file micromachines-13-01778-s001.zip › micromachines-1939250-supplementary.pdf]

Mathematical Model for Numerical Simulations of Polyelectrolyte-Surfactant Association in Microfluidic Channels

We will start with the analysis of a microchip with 2 inputs:

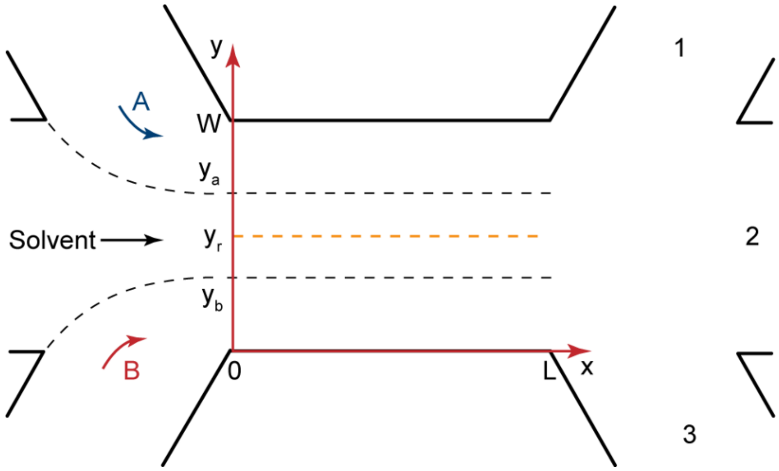

Figure SI1. Geometry of a microfluidic chip with the length L and the width W; A – surfactant, B – polymer; y_r is the radial coordinate of the reaction front.

A general second-order reaction equation described polyelectrolyte-surfactant complexation:

$$A + B \leftrightarrow C \tag{S1}$$

where B is the concentration of polyelectrolyte ionogenic groups, A is the concentration of surfactant ions, and C is the concentration of polyelectrolyte ionogenic groups with bound surfactant ions.

The rate law for surfactant is:

$$[A]'_{\tau} = -k_f[A][B] \tag{S2}$$

where k_f is the association rate constant. In this simplified mode, we neglect the reverse reaction of the complex dissociation because it will not be the governing factor for the emergence of a chemical signal.

If such a reaction occurs in a microfluidic channel, we need to add the diffusion term $D_A\Delta[A]$, according to Fick's second law:

$$[A]'_\tau = D_A\Delta[A] - k_f[A][B] \tag{S3}$$

We also need to add the convection term $U(y)\nabla[A]$, that describes the axial pressure-driven flow of the reacting species with a parabolic flow velocity profile in a rectangular microchannel:

$$[P]'_\tau + U(y)\nabla[A] = D_A\Delta[A] - k_f[A][B] \tag{S4}$$

where D_A is the diffusion coefficient of the surfactant ions, $U(y) = \frac{3}{2}U(1 - \left(1 - \frac{y}{W}\right)^2)$ is the flow velocity, ∇ is the Nabla operator Δ is the Laplacian operator, and x is the axial coordinate of a point in a microchannel.

To simplify this equation, consider only the axial convection of a pressure-driven laminar flow in a microchannel, only the radial diffusion of the reacting species and steady state conditions in the microchip [1,2]:

$$U(y)[A]'_x = D_A[A]''_{yy} - k_f[A][B] \tag{S5}$$

where x and y are axial and radial coordinates in the main channel (Fig. SI1), $[P]'_x$ and $[P]''_{yy}$ are the partial derivatives of polymer concentration in the main channel.

The Eq. (5) is a non-linear second-order partial differential equation with a reversible reaction as a source-sink term.

Similar equations are derived for the polymer B and the reaction product C, so we obtain a system of partial differential equations that characterize behavior of a reacting polyelectrolyte-surfactant system in a microchannel shown in Fig. SI1:

$$\begin{cases} U(y)[A]'_x = D_A[A]''_{yy} - k_f[A][B] \\ U(y)[B]'_x = D_B[B]''_{yy} - k_f[A][B] \\ U(y)[C]'_x = D_C[C]''_{yy} + k_f[A][B] \end{cases} \tag{S6}$$

where [B] is the molar concentration of monomer binding sites at the polyelectrolyte macromolecule, and [C] is the molar concentration of monomer binding sites that bound surfactant ions; D_B, and D_C are the diffusion coefficients of monomer binding sites (equal to that of the polymer macromolecule) and polymer-surfactant complexes.

The boundary conditions for the walls of the microchannel are derived from the assumption that the reaction species do not penetrate through them [1]. For surfactant:

$$\begin{cases} [A]'_y(y=0)=0 \\ [A]'_y(y=W)=0 \end{cases} \quad (S7)$$

same for the polymer B and complex C.

The boundary conditions for the junction of the input flows (x = 0) formalize that the concentration of a reagent is equal to the initial concentration in the incoming flow and is zero elsewhere, while the initial product concentration is zero [1]:

$$\begin{cases} [A](x=0,y)=\begin{cases}[A]^0, y \geq y_a \\ 0, y < y_a\end{cases} \\ [B](x=0,y)=\begin{cases}[B]^0, y \leq y_b \\ 0, y > y_b\end{cases} \\ [C](x=0,y)=0 \end{cases} \quad (S8)$$

The coordinates y_a and y_b can be set by the flowrates of the reagents and the solvent. If Q_a, Q_b, and Q_s are the polymer, surfactant, and solvent flow rates, respectively, then (for the geometry in Fig. SI2):

$$y_a = \frac{Q_b + Q_s}{\sum Q} W, y_b = \frac{Q_b}{\sum Q} W \quad (S9)$$

Dimensional Analysis of Convection-Diffusion-Reaction Equations

Let us introduce dimensionless parameters for the microchannel coordinates and concentrations of the reagents:

$$[A]^* = \frac{[A]}{[A]^0}, [B]^* = \frac{[B]}{[B]^0}, x^* = \frac{x}{L}, \text{ and } y^* = \frac{y}{W} \quad (S10)$$

where $[A]^0$ and $[B]^0$ are initial concentrations of the reagents, W and L are channel width and length, respectively.

From the reaction stoichiometry: $[C] = [A]^0 - [A]$, then:

$$\frac{[C]}{[A]^0} = 1 - [A]^* \tag{S11}$$

And we can introduce dimensionless concentration for a complexation reaction product:

$$[C]^* = \frac{[C]}{[A]^0} \tag{S12}$$

Consider the Hagen-Poiseuille parabolic flow profile in a rectangular microchannel. For the coordinates shown in Fig. SI1:

$$U(y) = \frac{3}{2}U(1 - (2y^* - 1)^2) \tag{S13}$$

Introducing the function $\beta = \frac{3}{2}(1 - (2y^* - 1)^2)$, we will finally get:

$$U(y) = \beta U \tag{S14}$$

Transform the equation for surfactant in the system (6) into the dimensionless form:

$$\beta U \frac{[A]^0}{L}[A]^{*\prime}_{x} = D_A \frac{[A]^0}{W^2}[A]^{*\prime\prime}_{yy} - k_f[A]^0[B]^0[A]^*[B]^* \tag{S15}$$

Divide this equation by $[A]^0$ and multiply by W^2/D_A:

$$\beta \frac{UW}{D_A}\frac{W}{L}[A]^{*\prime}_{x} = [A]^{*\prime\prime}_{yy} - \frac{W^2}{D_A}k_f[B]^0[B]^*[A]^* \tag{S16}$$

Introduce the ratio Z of initial concentrations of the reagents A and B:

$$Z = \frac{[B]^0}{[A]^0} \tag{S17}$$

Then:

$$\beta \frac{UW}{D_A}\frac{W}{L}[A]^{*\prime}_{x} = [A]^{*\prime\prime}_{yy} - \frac{W^2}{D_A}k_f Z[A]^0[A]^*[B]^* \tag{S18}$$

The coefficients in the resulting dimensionless equation depend only on the properties of one reagent: surfactant (diffusivity and initial concentration) and the association rate constant:

$$\beta \frac{UW}{D_A} \frac{W}{L} [A]^{*\prime}_{x} = [A]^{*\prime\prime}_{yy} - \frac{W^2}{D_A} k_f [A]^0 Z [A]^* [B]^* \tag{S19}$$

Several dimensionless similarity criteria appear in Eq. (19):

Peclet number (the ratio of convection and diffusion rates):

$$Pe = \frac{UW}{D_A} \tag{S20}$$

Normalized microchannel length (the ratio of microchannel length and width):

$$L_N = \frac{L}{W} \tag{S21}$$

Damköhler number (the ratio of the characteristic time of diffusion to the characteristic time of reaction) for a second order direct reaction:

$$Da = \left(\frac{W^2}{D_A}\right) / \left(\frac{1}{k_f [A]^0}\right) = \frac{W^2}{D_A} k_f [A]^0 \tag{S22}$$

Then:

$$\beta \frac{Pe}{L_N} [A]^{*\prime}_{x} = [A]^{*\prime\prime}_{yy} - Da Z [A]^* [B]^* \tag{S23}$$

All the dimensionless numbers are marked red.

The same dimensional analysis can be performed for the equations with the polymer B and the product C. For polymer:

$$\beta U \frac{[B]^0}{L} [B]^{*\prime}_{x} = D_S \frac{[B]^0}{W^2} [B]^{*\prime\prime}_{yy} - k_f [A]^0 [B]^0 [A]^* [B]^* \tag{S24}$$

To use the same dimensionless numbers calculated for surfactant S with the convection-diffusion-reaction equation for the polymer B, let us introduce the dimensionless ratio of their diffusion coefficients $D_N = \frac{D_B}{D_A}$.

Divide the Eq. (24) by $[B]^0$ and multiply by W^2/D_A:

$$\beta \frac{UW}{D_A}\frac{W}{L}[B]^{*\prime}_{x} = \frac{D_B}{D_A}[B]^{*\prime\prime}_{yy} - \frac{W^2}{D_A}k_f[A]^0[A]^*[B]^* \tag{S25}$$

Modify Eq. (27) by substituting $D_N = \frac{D_B}{D_A}$:

$$\beta \frac{UW}{D_A}\frac{W}{L}[B]^{*\prime}_{x} = D_N[B]^{*\prime\prime}_{yy} - \frac{W^2}{D_A}k_f[A]^0([A]^*[B]^* \tag{S26}$$

Finally:

$$\beta \frac{Pe}{L_N}[B]^{*\prime}_{x} = D_N[B]^{*\prime\prime}_{yy} - Da([A]^*[B]^* \tag{S27}$$

All the dimensionless numbers are marked red.

Dimensionless analysis of the equation for the product C:

$$\beta U \frac{[A]^0}{L}[C]^{*\prime}_{x} = D_C\frac{[A]^0}{W^2}[C]^{*\prime\prime}_{yy} + k_f[A]^0[B]^0[A]^*[B]^* \tag{S28}$$

To use the same dimensionless numbers calculated for polymer P with the convection-diffusion-reaction equations for the product C, let us introduce the dimensionless ratio of their diffusion coefficients $D_{CN} = \frac{D_C}{D_A}$.

Divide the Eq. (28) by $[A]^0$ and multiply by W^2/D_A:

$$\beta \frac{UW}{D_A}\frac{W}{L}[C]^{*\prime}_{x} = \frac{D_C}{D_A}[S]^{*\prime\prime}_{yy} + \frac{W^2}{D_A}k_f[B]^0[A]^*[B]^* \tag{S29}$$

Modify Eq. (27) by substituting $[B]^0 = \frac{1}{Z}[A]^0$ and $D_{CN} = \frac{D_C}{D_A}$:

$$\beta \frac{UW}{D_A}\frac{W}{L}[C]^{*\prime}_{x} = D_{CN}[C]^{*\prime\prime}_{yy} + \frac{W^2}{D_C}k_f[A]^0\frac{1}{Z}[A]^*[B]^* \tag{S30}$$

Finally:

$$\beta \frac{Pe}{L_N}[C]^{*\prime}_{x} = D_{CN}[C]^{*\prime\prime}_{yy} + \frac{Da}{Z}[A]^*[B]^* \tag{S31}$$

All the dimensionless numbers are marked red.

The final dimensionless system of equations, which will model an A + B ↔ C reaction in a microchannel:

$$\begin{cases} \beta \frac{Pe}{L_N}[A]_x^{*\prime} = [A]_{yy}^{*\prime\prime} - DaZ[A]^*[B]^* \\ \beta \frac{Pe}{L_N}[B]_x^{*\prime} = D_N[B]_{yy}^{*\prime\prime} - Da([A]^*[B]^* \\ \beta \frac{Pe}{L_N}[C]_x^{*\prime} = D_{CN}[C]_{yy}^{*\prime\prime} + \frac{Da}{Z}[A]^*[B]^* \end{cases} \tag{S32}$$

This system is non-dimensional and considers the parabolic flow velocity profile.

Naumann boundary conditions at microchannel walls in the dimensionless form:

$$\begin{cases} [A]_y^{*\prime}(y^* = 0) = 0 \\ [A]_y^{*\prime}(y^* = 1) = 0 \end{cases} \tag{S33}$$

same for the polymer B and complex C.

Dirichlet boundary conditions at the main channel input in the dimensionless form:

$$\begin{cases} [A](x^* = 0, y^*) = \begin{cases} [A]^0, y^* \geq y_a^* \\ 0, y^* < y_a^* \end{cases} \\ [B](x^* = 0, y^*) = \begin{cases} [B]^0, y^* \leq y_b^* \\ 0, y^* > y_b^* \end{cases} \\ [C](x^* = 0, y^*) = 0 \end{cases} \tag{S34}$$

Where $y_a^* = \frac{y_a}{W}$, $y_b^* = \frac{y_b}{W}$, and $y_r^* = \frac{y_r}{W}$

References

1. Berthier, J.; Silberzan, P., Microfluidics for Biotechnology, Second Edition. Artech House: London, 2009; p 512.
2. Tabeling, P., Introduction to Microfluidics. Oxford University Press: 2005; p 312.

The results of numerical modeling of polyelectrolyte-surfactant complexation in the main channel of a 3-input microfluidic chip with the central flow of solvent

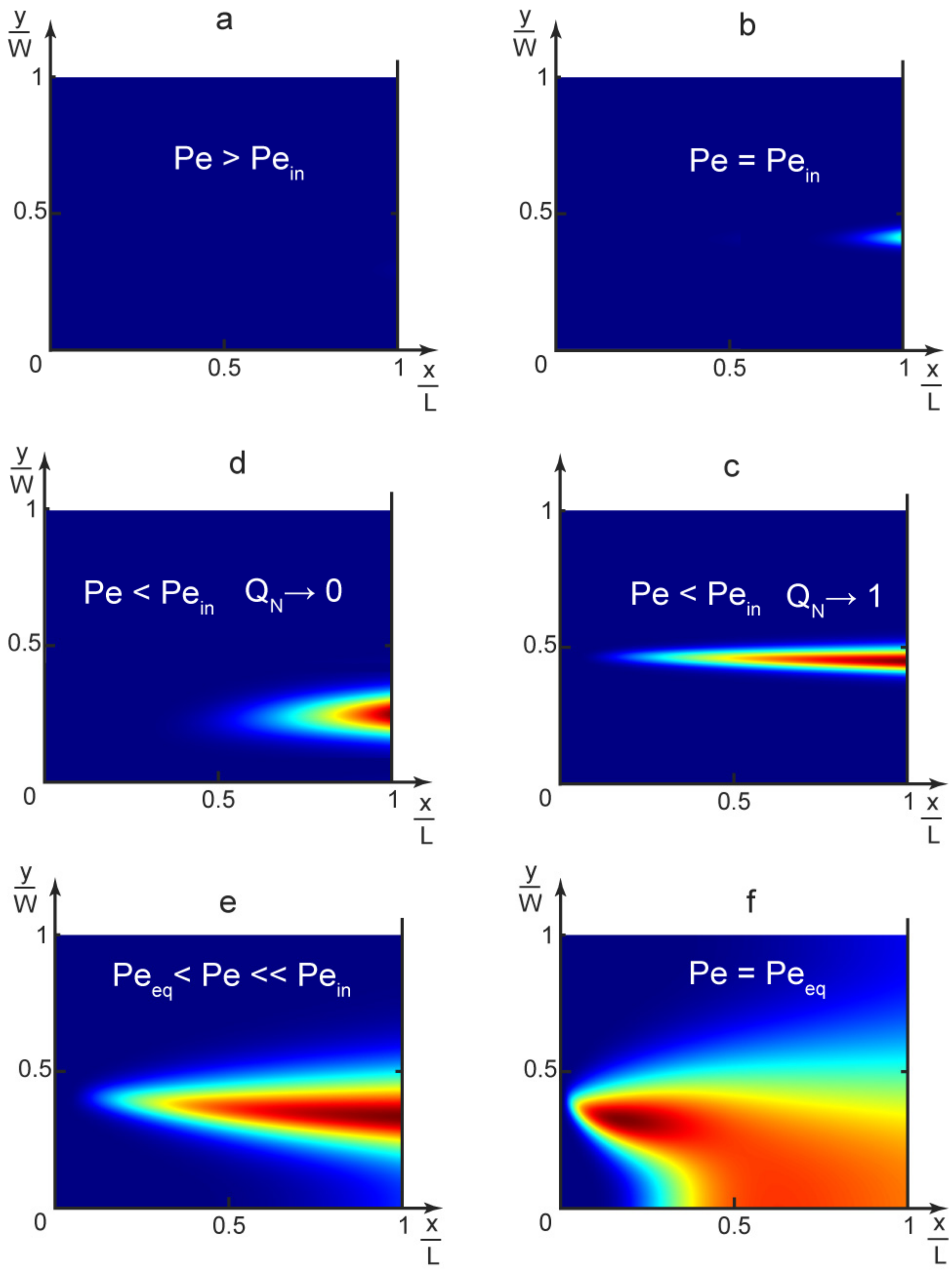

Figure SI2. Numerical simulations results at the main channel (x = L, y = W) for the PDADMAC-SDS complex concentration field. Flow rates: a) A, B – 5 µl/min, solvent – 10 µl/min; b) A, B – 5 µl/min, solvent – 3 µl/min; c) A, B – 0.2 µl/min, solvent – 1.2 µl/min; d) A, B – 10 µl/min, solvent

– 2 μl/min; e) A, B, solvent – 1 μl/min; d) A, B, solvent – 0.1 μl/min. The main channel width = 300 μm and length = 15 mm.
